# Disability weights for environmental noise-related health states: results of a disability weights measurement study in Europe

**DOI:** 10.1136/bmjph-2023-000470

**Published:** 2024-04-19

**Authors:** Periklis Charalampous, Carolien C H M Maas, Juanita A Haagsma

**Affiliations:** 1 Department of Public Health, Erasmus MC University Medical Center, Rotterdam, The Netherlands

**Keywords:** public health, epidemiology, noise

## Abstract

**Introduction:**

Measurement of the burden of disease using disability-adjusted life years requires disability weights to quantify health losses for non-fatal consequences of disease and injury. We aimed to obtain a set of disability weights for environmental and non-environmental noise-related health states (NOISE) using a nationally representative sample survey among the general population of four European countries; and to compare the resulting NOISE disability weights with those estimated in the Global Burden of Disease 2010 (GBD 2010) and European (EURO) disability weights measurement studies.

**Methods:**

We administered a web-based survey among a cohort of individuals from Hungary, Italy, Sweden and the Netherlands. It included paired comparison questions on 82 different health states. Each respondent performed 13 paired comparison tasks. We analysed paired comparison responses with probit regression analysis, and regression results were anchored on the disability weight scale between 0 (equivalent to full health) and 1 (equivalent to death).

**Results:**

In total, 4056 respondents participated in the study. Comparison of the regression results from paired comparison responses for each country-specific dataset with those run on the pooled dataset showed high linear correlations (0.96–0.98, p<0.001). The resulting disability weights ranged from 0.005 for mild impairment of distance vision and mild anaemia to 0.761 for intensive care unit admission. The disability weight for moderate and severe annoyance was 0.006 and 0.011, respectively. Comparison of disability weights showed a higher correlation between EURO and NOISE disability weights (pseudo R-squared=0.955, Pearson correlation=0.954) compared with GBD 2010 and NOISE disability weights (pseudo R-squared=0.893, Pearson correlation=0.946).

**Conclusions:**

The NOISE disability weights are consistent and highly correlated across the four European countries. The NOISE disability weights set can be used to estimate the burden of disease attributable to noise-related outcomes across Europe.

**WHAT IS ALREADY KNOWN ON THIS TOPIC**

- Assessment of the burden of disease expressed in disability-adjusted life years (DALYs) requires disability weights to quantify health losses for non-fatal consequences of disease and injury.
- The Global Burden of Disease 2010 (GBD 2010) disability weights measurement study elicited disability weights for 220 health states, but did not assess disability weights for unique environmental noise-related health states such as noise annoyance and sleep disturbance.

**WHAT THIS STUDY ADDS**

- The current study adds to the existing scientific literature by creating a set of 82 disability weights for health states specifically related to environmental noise and those that are not (related to noise) based on respondents from four European countries (Hungary, Italy, Sweden and the Netherlands).
- The current study made a comparison of the resulting disability weights with the GBD 2010 and European disability weights.
- The resulting set of disability weights can be used to quantify the environmental noise-related burden of disease expressed in DALYs.

**HOW THIS STUDY MIGHT AFFECT RESEARCH, PRACTICE OR POLICY**

- The resulting disability weights allow for comparison to other environmental disease burden estimates since they were derived based on methodological design choices aligned with existing disability weights measurement studies.
- We found evidence that adding information about the environmental source of sleep disturbance to the health state description resulted in inconsequential differences in the health state valuation.

## Introduction

 Disability-adjusted life years (DALYs) are widely used to estimate the population health impact of diseases, injuries and risk factors, by summarising fatal and non-fatal loss of healthy time into a single figure. Non-fatal losses of healthy life, defined as years lived with disability (YLDs), are calculated as the number of people living with a particular health outcome multiplied by the disability weight.^1 2^ The disability weight reflects the relative severity of health losses associated with that outcome. It is measured on a scale between 0 and 1, with 0 indicating a state equivalent to full health and 1, a state equivalent to death.^3 4^

Disability weight estimations are based on health state valuations obtained from a group of individuals (eg, people from the general populations or health experts). However, the design choices for eliciting these health state valuations in order to arrive at disability weights have been debated.^5^,^9^ After the publication of the Global Burden of Disease 2010 (GBD 2010) disability weights measurement study,^10^ there has been a shift to using samples of the general population rather than panels of health experts and/or conceptually less complex techniques such as paired comparison, rather than, for instance, trade-offs.^11^ This has resulted in more disability weights studies applying the same design choices, as well as an increased comparability of disability weights.^11 12^ Several studies, applying the same methods as the GBD 2010 disability weights measurement study,^10^ have been carried out either to develop national-based sets of disability weights or to estimate disability weights, not available from the GBD study.^13^,^17^ For example, the European (EURO) disability weights measurement study estimated disability weights for 43 health states for which no disability weights were available.^13^

Up to now, disability weights for environmental noise-related health states, such as noise annoyance and sleep disturbance, derived based on the GBD 2010 disability weights methods have not been available.^10 18^ In Europe, environmental noise is estimated to contribute to over 45 000 new cases of ischaemic heart disease and to cause approximately 12 000 premature deaths per year.^19 20^ It has also been negatively associated with schoolchildren’s learning and cognitive performance, as well as with hearing impairments, sleep disturbance, tinnitus and other health problems.^21^,^26^ Furthermore, several DALY-calculation studies have shown that YLDs due to noise annoyance and sleep disturbance accounted for the majority of the total DALYs attributable to environmental noise.^27^,^32^ Most of these DALY-calculation studies have however discussed that the DALY uncertainty range related to noise annoyance and sleep disturbance is primarily due to variations in available disability weights as well as to the diverse methodological design choices used in their derivation. This highlights the need to derive disability weights for noise-related health states, such as noise annoyance and sleep disturbance, using standardised methods; thus, allowing for the comparability of noise-related DALY estimates with those associated with other health outcomes. The availability of environmental noise-related disability weights obtained via a standardised approach is essential for the estimation of health losses associated with environmental noise-related outcomes. Knowledge of the impact of environmental noise on different diseases may help informing decision-making regarding resource allocation and intervention strategies.

Against this background, the WHO Regional Office for Europe set out to update the existing disability weights for environmental noise-related health states. The aim of this study was to obtain a set of disability weights for environmental and non-environmental noise-related health states (NOISE) using a nationally representative sample survey among the general population of four European countries, and applying the same methods as the GBD 2010 disability weights measurement study. The resulting NOISE disability weights for the health states included in the GBD 2010 and EURO disability weights measurement studies were also compared.

## Methods

### Study setting and design

We performed a cross-sectional observational study in which a web-based survey was administered to a cohort of individuals from the general population of Hungary, Italy, Sweden and the Netherlands. The survey questionnaire was translated from English into Dutch, Hungarian, Italian and Swedish using outsourced translation services and subsequently translated back into English. Bilingual native speakers verified the translations independently. Data were collected between 24 October 2022 and 9 November 2022. We followed methodological and analytical procedures aligned with the GBD 2010 disability weights measurement study.^10^ This study complies with the Strengthening the Reporting of Observational Studies in Epidemiology (STROBE) statement (online supplemental appendix, p17).^33^

### Panel participants and eligibility criteria

Participants were recruited by Flycatcher Internet Research, which distributed and launched the questionnaire. Samples were drawn from existing internet panels consisting of members of the general population residing in the above countries. These four countries were chosen as they are believed to be geographically representative of the European Union and European Economic Area member states.^13 14^

Participants aged 18–75 years, who had sufficient command of the native language of the country of residency were included. To ensure national representativeness, we preselected Dutch panel participants based on age, gender and highest attained level of education; such individual characteristics were already known for the Dutch panel participants. As for the selection of participants from Italy, Hungary and Sweden, a hard-quota sampling method based on age and gender (crossed) and education level (not crossed) was used.

### Health states lay descriptions

In total, 82 health states were evaluated. The selection of these health states began with identification of environmental noise-related health states, which was done through a scoping literature search (online supplemental appendix, pp 2). The selection of these health states ended with identification of non-environmental noise-related health states to ensure that the whole severity spectrum of health states was covered and to evaluate the validity and comparability of the resulting NOISE disability weights.

The NOISE disability weights set consisted of 44 health states that were included in both GBD 2010 and EURO disability weights studies with unmodified lay descriptions^10 13^; 14 health states from the GBD 2010 disability weights study for which modified health state descriptions were included in the EURO disability weights study^10 13^; 12 health states that were included in the GBD 2010 disability weights study but not in the EURO disability weights study^10 13^; 3 health states that were included in the EURO disability weights study but not in the GBD 2010 disability weights study^10 13^; and 9 new health states for which new lay descriptions were developed.

We constructed the brief lay descriptions (ie, up to 70 words; simple and non-clinical vocabulary) for the nine new health states after consultation with the Coordination Group, convened by the WHO Regional Office for Europe, and using the same design principles used in the GBD 2010 disability weights measurement study.^10^ All new health state descriptions were checked by an experienced physician and then, translated by professional outsourced translation services, and independently back-translated by bilingual native speakers. The health states and lay descriptions in English are presented in full in the (online supplemental appendix, table 1, p 4).

### Valuation of health states and data quality control

We used the paired comparison technique to elicit health state valuations for all 82 health states. With this technique, respondents need to consider two hypothetical individuals (person A vs person B) with different health states and to specify which person they regarded as healthier over the other. Each respondent performed 13 paired comparison tasks which were drawn randomly (using a computer-generated algorithm) from all available possible comparisons. A sample paired comparison question for two random environmental or non-environmental health states is shown in box 1 and in the online supplemental appendix (p 20).

Box 1 **A sample paired comparison question**

Imagine that both people will have these problems for the rest of their lives. Who do you think is healthier overall, the person A or the person B?

- Person A has severe pain, extreme fatigue, weight loss and high anxiety.
- Person B has chest pain that occurs with strenuous physical activity, such as running or lifting heavy objects. After a brief rest, the pain goes away.

We considered a series of measures to improve data quality. First, the data capture system did not allow for missing values, meaning that participants were required to respond to all questions. Second, the data capture system did not allow participants to adjust their responses (ie, go back in the questionnaire), so as to limit the response burden and enhance internal consistency. Third, we repeated the same pair of health states in the 2nd and 13th paired comparison questions, with health states presented in the same order (2nd question) and reverse order (13th question), so as to allow assessment of internal consistency and test–retest reliability of paired comparison responses.

## Sociodemographic characteristics

This study’s web survey also included questions on sociodemographic attributes such as age, gender, highest education level achieved, income, occupational status, environmental noise annoyance, living situation and self-reported chronic conditions. First, the educational level of each respondent was determined using the International Standard Classification of Education (ISCED-2011) and categorised into three groups: low (ISCED: 0, 1 and 2), middle (ISCED: 3 and 4) and high (ISCED: 5 and/or higher). Second, chronic conditions status was determined by the presence of several conditions (eg, asthma or chronic bronchitis, heart disease, stroke consequences, diabetes, rheumatoid arthritis, severe back complaints, cancer, memory problems due to ageing, memory problems due to dementia, depression or anxiety disorder, etc). Chronic condition status was categorised into five groups: 0, 1, 2, 3 and 4 or more. Third, self-reported environmental noise annoyance was determined using the International Commission on Biological Effects of Noise (ICBEN) standardised general purpose noise reaction question: ‘*Thinking about the last 12 months, when you are here at home, how much does environmental noise bother, disturb or annoy you? (eg, noise from air, road and rail traffic, industrial activity)’*.^34^ The noise annoyance responses were then categorised into four groups: not at all, slightly, moderately and highly (ie, scale point ‘very’ and ‘extremely’) annoyed by noise.^35^ The decision to combine the top two response categories (ie, the upper 40% of the verbal 5-point scale) was made in order to measure the proportion of respondents who are highly annoyed by noise.^34 35^

## Statistical analysis

We analysed paired comparison responses based on the choice probabilities over all possible pairs of health states—that is, the probability that the first health state in a pair was chosen as being the healthier over the other. Response probabilities for all observations were ordered in a matrix and plotted using a heat map. Each cell in the heat map indicates the response probability for one pair of health states. The colours of the heat map correspond to the probability that the first health state in a pair comparison is chosen as the healthier outcome. Reliability of the paired comparison responses was tested in the form of a test–re-test analysis.

Furthermore, we ran probit regression analyses on the paired comparison responses in pooled and country-specific datasets. A binary response variable was given a value of 1 if the first health state of the pair was selected as the healthier one; a value of −1 if the second health state of the pair was selected as the healthier; and 0 for all health states other than the pair being considered. The Pearson correlation coefficient was used to evaluate the relation within country-specific coefficients, as well as between country-specific and pooled regression coefficients. For instance, a high Pearson correlation (>0.80) between country-specific and pooled regression coefficients indicates that country-specific results are highly correlated and thus, well reflected by the pooled results.

To predict NOISE disability weights, we ran a non-parametric regression model (loess) of the logit-transformed disability weights derived from the GBD 2010 disability weights measurement study against the pooled regression coefficients. To quantify the goodness-of-fit of the non-parametric regression model, we used the proportion of explained variance (pseudo R-squared). We then simulated 1000 bootstrap samples with means defined by the predicted probit coefficients and variance by the SD of the predicted probit coefficients. On each bootstrap sample, we fitted a non-parametric model of the logit-transformed disability weights derived from the GBD 2010 disability weights set against the pooled regression coefficients. An inverse logistic transformation was applied to the mean predicted disability weights in order to obtain the NOISE disability weights on a 0–1 scale. Finally, the 95% uncertainty intervals (95% UI) were obtained from the corresponding distribution of the sampled disability weights.

We compared the resulting disability weights with those estimated in the GBD 2010 and EURO disability weights studies to check for consistency using the pseudo R-squared approach. Briefly, the goal of this approach is to assess the goodness-of-fit of the non-parametric regression model, that is, how well can the resulting disability weights be explained based on the existing sets of disability weights. A pseudo R-squared value close to 1 indicates a well-fitted model and might give an indication that the predictions of the NOISE disability weights are valid. Finally, we compared the resulting disability weights for those health states included in the GBD 2010 and EURO disability weights sets by calculating the absolute difference between GBD 2010 and NOISE disability weights as well as between EURO and NOISE disability weights. We plotted the top 10 health states with the largest differences. All analyses were performed with R (V.4.1.0) and SPSS (V.28.0.1).

## Results

### Study population

**Figure 1:**
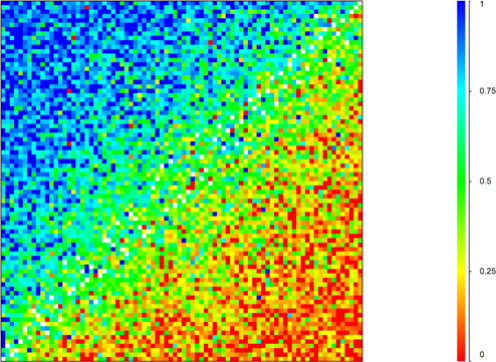
Response probabilities for paired comparisons.Red corresponds to less than 0.25. Orange, yellow and green correspond to probabilities between 0.25 and 0.75. Blue corresponds to probabilities greater than 0.75. Please note that not all possible 82×82 pairs were evaluated by pairwise comparison, which is indicated by some white spaces in the figure.

**Figure 2:**
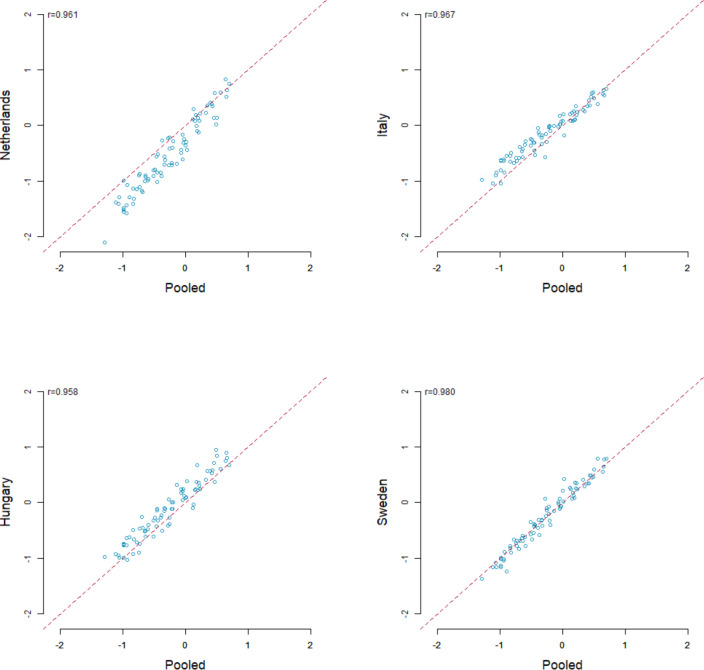
Country-specific regression results compared with pooled regression results for paired comparisons.

Table 1 shows the characteristics of the respondents. In total, 4056 respondents completed the questionnaire; 1028 from the Netherlands, 1026 from Sweden, 1002 from Italy and 1000 from Hungary. The median age of all respondents was 49 (IQR: 28). Slightly more than half the respondents were females (53.3%). Around 78% of the respondents had a middle or high educational level, and approximately half (48.9%) had no chronic conditions. Out of all the participants, 38% were not at all annoyed by noise, whereas 8.3% were highly annoyed (table 1). As this questionnaire was conducted on a convenience sample, we could not calculate a response rate. The distribution of gender, age groups and educational level of the NOISE cohort sample per country is presented in the online supplemental appendix (figure 1), p10. The distribution of gender and age groups of the NOISE cohort sample versus the national population per country is also presented in the online supplemental appendix (figure 2), p11.

**Table 1:** Characteristics of the study population

		The Netherlands	Italy	Hungary	Sweden	Total
		**n=1028**	**n=1002**	**n=1000**	**n=1026**	**n=4056**
Age	Median (IQR)	51 (29)	48 (23)	47 (26)	51 (32)	49 (28)
Age group	18–34 years	265 (25.8%)	223 (22.3%)	246 (24.6%)	285 (27.8%)	1019 (25.1%)
	35–54 years	336 (32.7%)	394 (39.3%)	370 (37.0%)	281 (27.4%)	1381 (34.0%)
	55–75 years	427 (41.5%)	385 (38.4%)	384 (38.4%)	460 (44.8%)	1656 (40.8%)
Gender	Male	523 (50.9%)	476 (47.5%)	460 (46.0%)	425 (41.4%)	1884 (46.4%)
	Female	505 (49.1%)	523 (52.2%)	539 (53.9%)	596 (58.1%)	2163 (53.3%)
	Other	0 (0%)	3 (0.3%)	1 (0.1%)	5 (0.5%)	9 (0.2%)
Education level	Low	247 (24.0%)	231 (23.1%)	204 (20.4%)	216 (21.1%)	898 (22.1%)
	Middle	451 (43.9%)	500 (49.9%)	481 (48.1%)	350 (34.1%)	1782 (43.9%)
	High	330 (32.1%)	271 (27.0%)	315 (31.5%)	460 (44.8%)	1376 (33.9%)
Occupation status	Employed	642 (62.5%)	536 (53.5%)	625 (62.5%)	476 (46.4%)	2279 (56.2%)
	Unemployed	38 (3.7%)	150 (15.0%)	57 (5.7%)	82 (8.0%)	327 (8.1%)
	Student	64 (6.2%)	65 (6.5%)	32 (3.2%)	63 (6.1%)	224 (5.5%)
	Retired	192 (18.7%)	177 (17.7%)	225 (22.5%)	308 (30.0%)	902 (22.2%)
	Unable to work	73 (7.1%)	11 (1.1%)	16 (1.6%)	84 (8.2%)	184 (4.5%)
	Other	19 (1.8%)	63 (6.3%)	45 (4.5%)	13 (1.3%)	140 (3.5%)
Household income	Low	316 (30.7%)	141 (14.1%)	308 (30.8%)	487 (47.5%)	1252 (30.9%)
	Middle	149 (14.5%)	441 (44.0%)	345 (34.5%)	109 (10.6%)	1044 (25.7%)
	High	362 (35.2%)	319 (31.8%)	252 (25.2%)	312 (30.4%)	1245 (30.7%)
	Unwilling to tell or do not know	201 (19.6%)	101 (10.1%)	95 (9.5%)	118 (11.5%)	515 (12.7%)
Chronic conditions	0	545 (53.0%)	553 (55.2%)	469 (46.9%)	417 (40.6%)	1984 (48.9%)
	1	292 (28.4%)	289 (28.8%)	267 (26.7%)	329 (32.1%)	1177 (29.0%)
	2	119 (11.6%)	95 (9.5%)	144 (14.4%)	164 (16.0%)	522 (12.9%)
	3	41 (4.0%)	43 (4.3%)	60 (6.0%)	69 (6.7%)	213 (5.3%)
	4 or more	31 (3.0%)	22 (2.2%)	60 (6.0%)	47 (4.6%)	160 (3.9%)
Living situation	Not living alone	813 (79.1%)	891 (88.9%)	769 (76.9%)	676 (65.9%)	3149 (77.6%)
	Living alone	209 (20.3%)	101 (10.1%)	205 (20.5%)	342 (33.3%)	857 (21.1%)
	Other	6 (0.6%)	10 (1.0%)	26 (2.6%)	8 (0.8%)	50 (1.2%)
Noise annoyance (eg, air traffic, road traffic, rail traffic and industrial activity)	Not at all	419 (40.8%)	225 (22.5%)	421 (42.1%)	475 (46.3%)	1540 (38.0%)
	Slightly	438 (42.6%)	364 (36.3%)	324 (32.4%)	265 (25.8%)	1391 (34.3%)
	Moderately	113 (11.0%)	276 (27.5%)	204 (20.4%)	193 (18.8%)	786 (19.4%)
	Highly	58 (5.6%)	137 (13.7%)	51 (5.1%)	93 (9.1%)	339 (8.3%)

### Paired comparison responses

**Figure 3:**
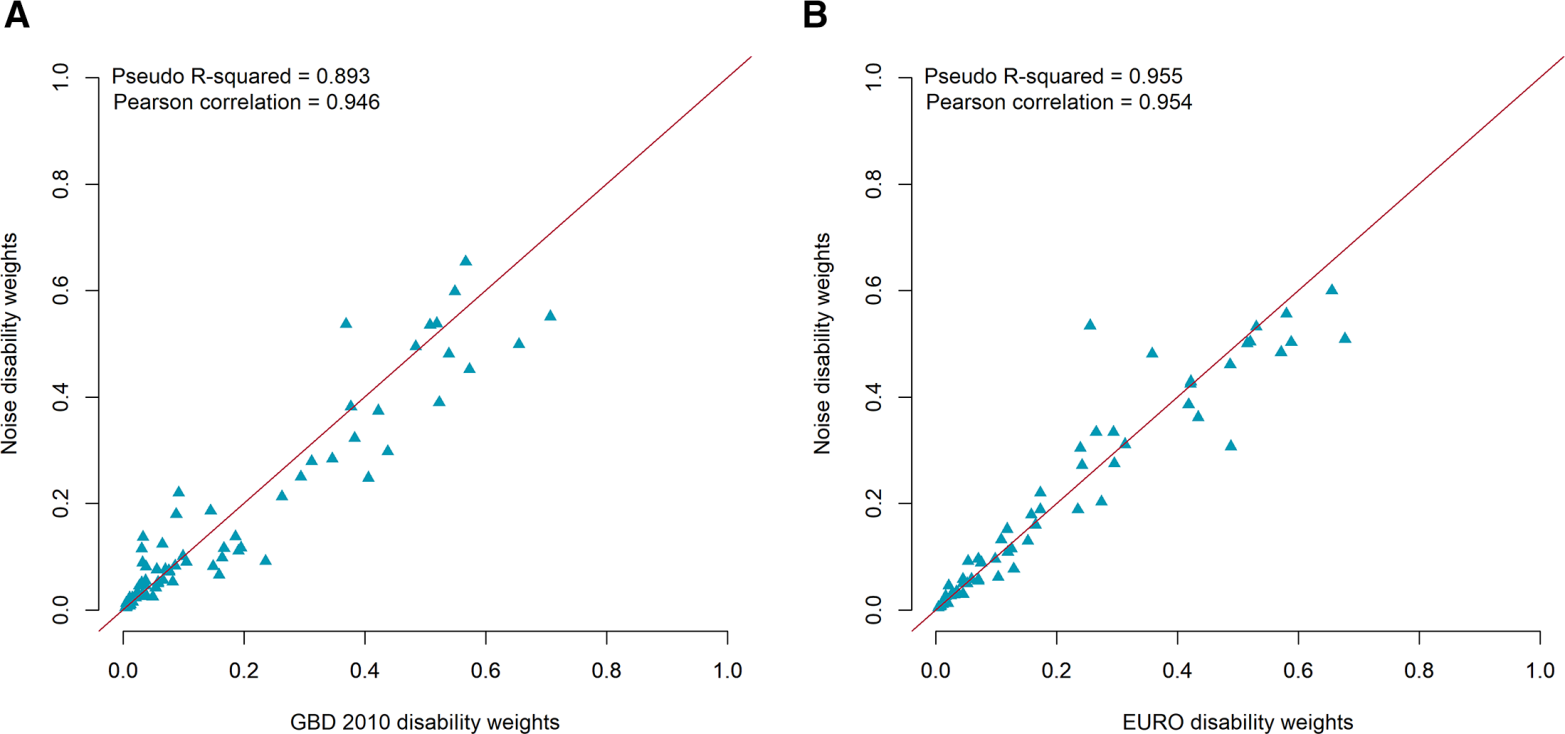
Comparison of NOISE disability weight estimates to those estimated in (A) GBD 2010 disability weights and (B) EURO disability weights studies.

Figure 1 illustrates a heat map of the paired comparison response probabilities for the 82×82 possible paired comparisons. The heat map of paired comparison response probabilities for all possible paired comparisons shows a smooth transition in colours from high (upper left) to low (lower right) probabilities (figure 1). This indicates low measurement error as well as high internal consistency. All participants were given the same pair in the 2nd and 13th paired comparison task, and of these 50.9% were presented in the same order, and 49.1% in reverse order. In all countries, the probability of choosing the same health state was higher if the two health states were presented in the same order (0.78) compared with reverse order (0.73). The probability of choosing the same health state was higher among highly educated (0.77) compared with low-educated (0.74) participants. Test–retest analysis by country and educational level and heat maps of the paired comparison probabilities per country are provided in the online supplemental appendix (figure 3), p13.

Comparison of the regression coefficients for each country-specific dataset with those run on the pooled dataset showed high linear correlations. Pearson correlation coefficients between country-specific and pooled regression analyses were 0.95 or higher (figure 2), whereas within country-specific regression analyses, they were 0.85 or higher (online supplemental appendix (figure 6), p16). The highest correlation of the probit coefficients between country-specific and pooled results was observed in Sweden (r=0.980, p<0.001) and the lowest in Hungary (0.958, p<0.001) (figure 2). The Pearson correlation coefficients within country-specific results ranged from 0.860 (p<0.001) in the Netherlands versus Hungary to 0.939 (p<0.001) in the Netherlands versus Sweden (online supplemental appendix (figure 6), p 16).

## Disability weights

Table 2 shows the resulting NOISE disability weights and 95% UI. In total, 29 out of 82 (35%) health states had a disability weight lower than 0.05. Mild impairment of distance vision (0.005) and mild anaemia (0.005) had the lowest disability weight, whereas intensive care unit admission (0.761) followed by severe long-term consequences of stroke plus cognitive problems (0.654) had the highest disability weight. We identified a high level of face validity between the estimated disability weights; the disability weight values increased by level of severity. For instance, mild heart failure had a disability weight of 0.046; moderate heart failure had a disability weight of 0.076; and severe heart failure had a disability weight of 0.138. A similar pattern of agreement was observed between health states consisting of two severity levels; that is, moderate annoyance had a lower disability weight (0.006) compared with severe annoyance (0.011). The disability weight for sleep disturbance without environmental noise as the source was 0.009 and disability weight for sleep disturbance with environmental noise as the source was 0.010 (table 2).

**Table 2:** Disability weights for 82 health states

	Disability weight (95% UI)
**Cancer**	
Diagnosis and primary therapy	0.254 (0.167–0.359)
Metastatic	0.495 (0.376–0.627)
Mastectomy	0.081 (0.059–0.106)
Stoma	0.084 (0.062–0.110)
Terminal phase, with medication (for cancers, end-stage kidney/liver disease)	0.535 (0.416–0.666)
Terminal phase, without medication (for cancers, end-stage kidney/liver disease)	0.538 (0.422–0.669)
**Cardiovascular and circulatory disease**	
Acute myocardial infarction	
Days 1–2	0.374 (0.267–0.492)
Days 3–28	0.076 (0.055–0.101)
Angina pectoris	
Mild	0.055 (0.037–0.076)
Moderate	0.056 (0.038–0.077)
Severe	0.116 (0.081–0.166)
Cardiac conduction disorders and cardiac dysrhythmias	0.186 (0.119–0.272)
Claudication	0.015 (0.010–0.022)
Heart failure	
Mild	0.046 (0.031–0.065)
Moderate	0.076 (0.055–0.100)
Severe	0.138 (0.092–0.204)
Stroke	
Long-term consequences	
Mild	0.023 (0.015–0.033)
Moderate	0.072 (0.052–0.096)
Moderate plus cognitive problems	0.279 (0.188–0.384)
Severe	0.481 (0.364–0.611)
Severe plus cognitive problems	0.654 (0.510–0.796)
**Diabetes, digestiveand genitourinary disease**	
Diabetic foot	0.032 (0.021–0.046)
Diabetic neuropathy	0.101 (0.074–0.137)
Chronic kidney disease (stage IV)	0.090 (0.067–0.120)
End-stage renal disease with kidney transplant	0.026 (0.017–0.038)
End-stage renal disease on dialysis	0.452 (0.335–0.576)
Infertility	
Primary	0.008 (0.004–0.012)
Secondary	0.006 (0.003–0.010)
**Chronic respiratory diseases**	
Asthma	
Controlled	0.011 (0.007–0.017)
Partially controlled	0.046 (0.035–0.058)
COPD and other chronic respiratory disease	
Mild	0.023 (0.015–0.034)
Severe	0.323 (0.222–0.436)
**Neurological disorders**	
Dementia	
Mild	0.053 (0.036–0.075)
Moderate	0.284 (0.190–0.389)
Severe	0.298 (0.201–0.407)
Multiple sclerosis, severe	0.551 (0.425–0.696)
Epilepsy	
Severe (seizures ≥once per month)	0.615 (0.480–0.772)
Less severe (seizures <once per month)	0.218 (0.133–0.320)
Parkinson’s disease	
Mild	0.023 (0.015–0.034)
Moderate	0.215 (0.137–0.310)
Severe	0.599 (0.469–0.740)
**Mental, behaviouraland substance use disorders**	
Anxiety disorders	
Mild	0.026 (0.017–0.038)
Moderate	0.082 (0.060–0.108)
Severe	0.390 (0.281–0.509)
Major depressive disorder	
Mild episode	0.066 (0.046–0.089)
Moderate episode	0.248 (0.161–0.347)
Severe episode	0.499 (0.375–0.637)
Attention-deficit hyperactivity disorder	0.025 (0.016–0.036)
Conduct disorder	0.092 (0.068–0.123)
**Hearing and vision loss**	
Hearing loss	
Mild	0.013 (0.008–0.020)
Moderate	0.027 (0.018–0.040)
Severe	0.089 (0.066–0.118)
Profound	0.115 (0.080–0.169)
Complete	0.137 (0.092–0.205)
Mild with ringing	0.028 (0.018–0.041)
Moderate with ringing	0.050 (0.033–0.070)
Severe with ringing	0.124 (0.086–0.178)
Profound with ringing	0.180 (0.114–0.270)
Complete with ringing	0.220 (0.142–0.317)
Distance vision	
Mild impairment	0.005 (0.002–0.009)
Moderate impairment	0.030 (0.020–0.045)
Severe impairment	0.111 (0.079–0.156)
Blindness	0.117 (0.082–0.167)
**Other**	
Anaemia	
Mild	0.005 (0.003–0.009)
Moderate	0.052 (0.035–0.073)
Severe	0.098 (0.071–0.134)
Annoyance	
Moderate	0.006 (0.003–0.010)
Severe	0.011 (0.006–0.016)
Cognitive impairments	
Mild	0.013 (0.008–0.019)
Moderate	0.080 (0.059–0.106)
Severe	0.096 (0.070–0.131)
Generic uncomplicated disease	
Anxiety about diagnosis	0.042 (0.028–0.061)
Worry and daily medication	0.051 (0.035–0.071)
Intensive care unit admission	0.761 (0.492–0.946)
Loss of smell/taste	0.017 (0.011–0.025)
Motor impairment	
Mild	0.010 (0.006–0.016)
Moderate	0.074 (0.053–0.098)
Severe	0.382 (0.276–0.500)
Sleep disturbance without environmental noise as the source	0.009 (0.006–0.014)
Sleep disturbance with environmental noise as the source	0.010 (0.006–0.015)
Spinal cord lesion at neck level: treated	0.536 (0.408–0.666)
Tinnitus	0.044 (0.028–0.063)

### Comparison of NOISE disability weights with existing sets of disability weights

The pseudo R-squared between regression results of the EURO and NOISE disability weights was higher (pseudo R-squared=0.955, Pearson correlation=0.954) compared with the one calculated between the GBD 2010 and NOISE disability weights (pseudo R-squared=0.893, Pearson correlation=0.946) (figure 3). Most of the new health states were located at the lower end of the disability weight scale with values lower than 0.2 (eg, tinnitus, annoyance, sleep disturbance, cognitive impairments and loss of smell/taste). Overall, some health states in the NOISE disability weights set had lower disability weight compared with those estimated in the GBD 2010 and EURO disability weights studies. For instance, severe multiple sclerosis had a disability weight of 0.551 (NOISE disability weights set), whereas this health state had previously been estimated as being among the highest disability weight (severe multiple sclerosis; GBD 2010 disability weight=0.707 and EURO disability weight=0.677).

## Discussion

We aimed to derive disability weights for 82 health states based on health state valuations of nationally representative samples from four European countries. The resulting disability weights ranged from 0.005 for mild impairment of distance vision and mild anaemia to 0.761 for intensive care unit admission. We found a logical order in disability weights for all health states with multiple severity levels—that is, mild health states had lower values compared with moderate and severe health states. This is indicative of high face validity. This logical ordering of health states with different severity levels was also observed in the GBD 2010 and EURO disability weights studies.^10 13^

Some of the health states included in this study were also included in the GBD 2010 and EURO disability weights studies.^10 13^ Comparison of disability weights of these health states showed a high correlation between these studies, particularly between the EURO disability weights measurement study and this study.^13^ However, the actual disability weights derived from this study were slightly lower compared with the GBD 2010 disability weights measurement study.^10^ Since the evaluated health state descriptions and valuation method used were the same, an explanation for this finding may lie in a difference in the health state preferences of this study population versus the GBD 2010 disability weights measurement study.^10^ In this study, the population consisted of individuals from the general population of four European countries, whereas the GBD 2010 disability weights measurement study was based on health state valuations of individuals from more than 175 (small) geographical areas.

Previous studies have indicated that contextual differences may play an important role in health state valuations using paired comparisons^15 16^; these contextual factors may have resulted in differences in health state valuations and, therefore in slightly different disability weight values for the same health states. In particular, disability weight values that were derived based on responses of a Japanese versus Chinese versus European sample varied.^13^,^16^ For instance, in disability weights measurement studies conducted in China and Japan, it was observed that disability weights assigned to mental health symptoms were lower compared with those conducted in Europe.^13^,^16^ It was also found that disability weights for severe alcohol use disorder were lower in Fujian (China) than in Japan.^15 36^ Our findings did not indicate large contextual differences among our study participants. We found a high correlation within country-specific coefficients as well as between country-specific and pooled coefficients. This suggests that health state valuations based on paired comparisons are consistent across the European countries. This also confirms the results observed in the EURO disability weights measurement study where similar high correlations were observed.^13^ It is also possible to use the disability weights from our study in non-European region countries, but it is important that researchers keep in mind that these findings reflect the health state preferences of European populations and that, ideally, disability weights for noise-related health states are derived for the population under study.

Furthermore, the test–retest reliability of the paired comparison task was slightly higher (same order: 0.78; reversed order: 0.73) compared with the EURO disability weights measurement study^13^ (same order: 0.75; reversed order: 0.73), with little differences in test–retest reliability among individuals with different educational levels. This indicates that the quality of the paired comparison data was high and consistent across the educational levels of the respondents. The findings underline that the paired comparison technique is suitable for health state valuations in the general population, due to the low cognitive burden of the task, and possibly, also to the brevity of and lay terminology used in the descriptions of the health states.

Our study focused on environmental noise-related disability weights, such as noise annoyance and sleep disturbance. We estimated disability weights for moderate and severe annoyance to be 0.006 and 0.011, respectively. The disability weight for severe annoyance is much lower than the one (0.02) previously proposed by the WHO Regional Office for Europe^18 37^ and similar to that (0.01) proposed by van Kamp *et al*.^38^ An explanation for the former is that different methods for deriving disability weights result in different disability weight values.^12^ Considerable variations have been identified in health preferences and thus, in disability weights derived from, for instance, patients versus members of the general public or medical experts versus members of the general public.^39^,^42^ In fact, medical experts have greater understanding of or experience with specific medical conditions, which may result in different values for the same disability weights. In addition, the DALY metric explicitly views *health* as a vital population-based asset that enables individuals to live a long and healthy life, thereby making the integration of health preferences from (diverse) members of the general public vital. Furthermore, in this study a more detailed disaggregation of noise annoyance was used (ie, moderate and severe annoyance), whereas only one health state (ie, high annoyance) was previously considered.^18 37^ Future studies can therefore quantify environmental-related DALYs at both lower and higher granularity levels of noise annoyance, if epidemiological data exist.

Another noteworthy observation pertains to the difference between the disability weight for high sleep disturbance (HSD) estimated based on expert judgments and used by WHO (0.07)^18 37^ and the one estimated in this study which derived based on preferences from general populations (0.010). Nevertheless, the ranking of annoyance and sleep disturbance was similar across studies, with lower disability weights assigned to annoyance compared with sleep disturbance. Furthermore, our results indicate that including information about the environmental source of the symptoms and functional limitations described in the health state description resulted in inconsequential differences in disability weights (ie, disability weight for sleep disturbance: 0.009; 0.006–0.014 95% UI and disability weight for sleep disturbance with source: 0.010; 0.006–0.015 95% UI). Hence, one can assume that the same applies to noise annoyance and that information about the environmental source of a particular health state is not taken into account by participants when evaluating health states. However, it is strongly recommended to investigate this in future environmental noise-related disability weights measurement studies. It should also be noted that, additional information on functional impairments or symptoms was taken into account, as results from the EURO disability weights measurement study showed.^13^ In the EURO disability weights measurement study, two distinct health state descriptions for several health states with slight differences in the wording of functional impairments, resulted in, sometimes stark, differences in disability weights.^13^ In our study, we only investigated the impact of including environmental noise as the source of sleep disturbance. It remains to be investigated whether or not other sources of sleep disturbance (eg, smell or light exposure) also impact the evaluation of health states by the respondents.

A limitation of our study lies in the use of a web-based survey for data collection, which can be costly due to length of time required to collect data. However, computer-assisted personal interviews would probably have resulted in higher data quality, and delivered the most representative results. In addition, internet users are generally younger compared with the general population and highly educated individuals, with the Netherlands and Sweden having among the highest percentage of individuals with above-average basic overall digital skills in Europe, and Italy and Hungary having among the lowest compared with the European Union average.^43^ We sought to mitigate the above limitations by using (existing) large internet panels with regard to age, gender and highest level of education. Another limitation of our study lies in the formulation of lay health state descriptions for noise annoyance and sleep disturbance. The scarcity of information on the definition of noise annoyance and sleep disturbance versus insomnia as well as the lack of qualitative studies limit the development of precise lay descriptions for such noise-related health states. We sought to mitigate the above limitation by seeking opinion from medical experts as well as from environmental noise experts. However, one can argue that these lay descriptions need to be further refined, for example, by considering the functional limitations and/or psychological implications linked to these outcomes.^44 45^ Additionally, the WHO Environmental Noise Guidelines for the European Region and other studies have focused on (source-specific) exposure–response functions for noise-related HSD.^3746^,^48^ We estimated disability weight for sleep disturbance (ie, with and without environmental noise as the source) and not for %HSD. This should be taken into account when applying both the disability weight for sleep disturbance and exposure–response functions in environmental noise-related burden of disease studies. Notwithstanding the above limitations, the resulting European NOISE disability weights set is consistent, and can be used to estimate the environmental burden of disease attributable to noise-related health outcomes across Europe and beyond.

## supplementary material

10.1136/bmjph-2023-000470 online supplemental file 1

## Data Availability

Data are available upon reasonable request.
