# Supplementary material for: Disability weights for environmental noise-related health states: results of a disability weights measurement study in Europe
Source: BMJ Public Health. 2024 Apr 19;2(1):e000470. doi: 10.1136/bmjph-2023-000470 (PMC11812797; doi:10.1136/bmjph-2023-000470)
Supplement: online supplemental file 1 [file bmjph-2-1-s001.pdf]

Supplementary appendix to: *Disability weights for environmental noise-related health states: results of a disability weights measurement study in Europe*

Table of Contents

1. Identification of health states: a scoping literature search

2. Supplementary tables and figures

Table 1: Brief lay descriptions for 82 environmental and non-environmental noise-related health states

Figure 1: Distribution of gender, age groups, and educational level of the NOISE cohort sample, per country

Figure 2: Distribution of gender and age groups of the NOISE cohort sample *vs*. the national population, per country

Figure 3: Test re-test of the paired comparison, by country and educational level

Figure 4: Response probabilities for paired comparisons per country, and overall

Figure 5: Pearson correlation coefficients between country-specific and pooled regression analyses

Figure 6: Pearson correlation coefficients within country-specific regression analyses

3. STROBE Statement—checklist of items that should be included in reports of observational studies

4. European NOISE disability weights measurement study: questionnaire

1. Identification of health states: a scoping literature search

At a first step, we performed a scoping literature search of burden of disease studies that estimated disability-adjusted life years attributable to environmental noise-related outcomes. At a second step, we included non-environmental noise-related health states in order to ensure that the whole severity spectrum of health states is covered and to evaluate the validity and comparability of the resulting disability weights. Details of the search strategy can be found below.

The search consists of two searches for every database. The searches per database were merged with 'OR' (#1 OR #2). The endnote file that was prepared by the librarian includes the merged files.

Database searched	via	Years of coverage	Records	Records after duplicates removed
Embase	Embase.com	1971 - Present	1088	471
Medline ALL	Ovid	1946 - Present	808	806
Web of Science Core Collection*	Web of Knowledge	1975 - Present	309	56
**Total**			**2205**	**1333**

Embase.com (1) 231

('environmental noise'/de OR 'noise pollution'/exp OR 'noise exposure'/de OR noise/mj OR 'occupational noise exposure'/de OR 'noise injury'/de OR (((environment* OR pollut* OR traffic* OR aircraft* OR airplane* OR airport* OR aviation* OR industr* OR annoy* OR exposure* OR occupation* OR work* OR railway* OR railroad* OR road OR transport*) NEAR/3 noise*)):ab,ti OR noise:ti) AND (model/exp/mj OR 'disease model'/exp OR (((disease* OR develop*) NEAR/3 model*)):Ab,ti OR model*:ti) AND (neoplasm/exp OR 'diabetes mellitus'/exp OR 'cardiovascular disease'/de OR 'heart disease'/exp OR 'coronary artery disease'/exp OR 'disease model'/exp OR 'health status'/exp OR 'cerebrovascular disease'/exp OR 'disability-adjusted life year'/de OR mortality/exp OR morbidity/exp OR 'disease burden'/de OR 'cognitive defect'/exp OR (neoplas* OR cancer* OR diabet* OR coronar* OR heart OR cardiovascul* OR cerebrovascul* OR atri*-fibril* OR (disease* NEAR/3 model*) OR ((health OR mild OR moderate* OR sever* OR asymptom*) NEAR/3 (state* OR status OR outcome* OR impair*)) OR stroke* OR daly OR yll OR yld OR (disability*-adjust* NEAR/3 (life-year* OR life-year)) OR (year* NEAR/3 life-lost) OR (year* NEAR/3 (living OR lived) NEAR/3 disabilit*) OR mortalit* OR morbidit* OR sequelae* OR (cogniti* NEAR/3 (defect* OR impair*)) OR (burden* NEAR/3 disease*) OR dement*):Ab,ti) NOT ([animals]/lim NOT [humans]/lim) NOT [conference abstract]/lim

Embase.com (2) 874

('environmental noise'/mj/de OR 'noise pollution'/mj/exp OR 'noise exposure'/mj/de OR noise/mj OR 'occupational noise exposure'/mj OR 'noise injury'/mj OR (((environment* OR pollut* OR traffic* OR aircraft* OR airplane* OR airport* OR aviation* OR industr* OR annoy* OR exposure* OR occupation* OR work* OR railway* OR railroad* OR road OR transport*) NEAR/3 noise*)):ti) AND (neoplasm/mj/exp OR 'diabetes mellitus'/mj/exp OR 'cardiovascular disease'/mj OR 'heart disease'/exp/mj OR 'coronary artery disease'/exp/mj OR 'disease model'/mj/exp OR 'health status'/mj/exp OR 'cerebrovascular disease'/mj/exp OR 'disability-adjusted life year'/mj/de OR mortality/mj/exp OR morbidity/mj/exp OR 'disease burden'/mj/de OR 'cognitive defect'/exp/mj OR (neoplas* OR cancer* OR diabet* OR coronar* OR heart OR cardiovascul* OR cerebrovascul* OR atri*-fibril* OR (disease* NEAR/3 model*) OR ((health OR mild OR moderate* OR sever* OR asymptom*) NEAR/3 (state* OR status OR outcome* OR impair*)) OR stroke* OR daly OR yll OR yld OR (disability*-adjust* NEAR/3 (life-year* OR life-year)) OR (year* NEAR/3 life-lost) OR (year* NEAR/3 (living OR lived) NEAR/3 disabilit*) OR mortalit* OR morbidit* OR sequelae* OR (cogniti* NEAR/3 (defect* OR impair*)) OR (burden* NEAR/3 disease*) OR dement*):ti) NOT ([animals]/lim NOT [humans]/lim) NOT [conference abstract]/lim

Medline ALL (1) 120

(Noise, Occupational/ OR exp * Noise/ OR (((environment* OR pollut* OR traffic* OR aircraft* OR airplane* OR airport* OR aviation* OR industr* OR annoy* OR exposure* OR occupation* OR work* OR railway* OR railroad* OR road OR transport*) ADJ3 noise*)).ab,ti. OR noise.ti.) AND (exp * Models, Nursing/ OR (((disease* OR develop*) ADJ3 model*)).ab,ti. OR model*.ti.) AND (exp Neoplasms/ OR exp Diabetes Mellitus/ OR Cardiovascular Diseases/ OR exp Heart Diseases/ OR exp Coronary Artery Disease/ OR exp Health Status/ OR exp Cerebrovascular Disorders/ OR Disability-Adjusted Life Years/ OR exp Mortality/ OR exp Morbidity/ OR Cost of Illness/ OR (neoplas* OR cancer* OR diabet* OR coronar* OR heart OR cardiovascul* OR cerebrovascul* OR atri*-fibril* OR (disease* ADJ3 model*) OR ((health OR mild OR moderate* OR sever* OR asymptom*) ADJ3 (state* OR status OR outcome* OR impair*)) OR stroke* OR daly OR yll OR yld OR (disability*-adjust* ADJ3 (life-year* OR life-year)) OR (year* ADJ3 life-lost) OR (year* ADJ3 (living OR lived) ADJ3 disabilit*) OR mortalit* OR morbidit* OR sequelae* OR (cogniti* ADJ3 (defect* OR impair*)) OR (burden* ADJ3 disease*) OR dement*).ab,ti.) NOT (exp animals/ NOT humans/) NOT (conference abstract)

Medline ALL (2) 697

(* noise/OR * Noise, Occupational/ OR (((environment* OR pollut* OR traffic* OR aircraft* OR airplane* OR airport* OR aviation* OR industr* OR annoy* OR exposure* OR occupation* OR work* OR railway* OR railroad* OR road OR transport*) ADJ3 noise*)).ti.) AND (exp * Neoplasm/ OR exp * Diabetes Mellitus/ OR * Cardiovascular Diseases/ OR exp * Heart Diseases/ OR * Coronary Artery Disease/ OR exp * Health Status/ OR * Cerebrovascular Disorders/ OR * Disability-Adjusted Life Years/ OR exp * Mortality/ OR exp * Morbidity/ OR * Cost of Illness/ OR (neoplas* OR cancer* OR diabet* OR coronar* OR heart OR cardiovascul* OR cerebrovascul* OR atri*-fibril* OR (disease* ADJ3 model*) OR ((health OR mild OR moderate* OR sever* OR asymptom*) ADJ3 (state* OR status OR outcome* OR impair*)) OR stroke* OR daly OR yll OR yld OR (disability*-adjust* ADJ3 (life-year* OR life-year)) OR (year* ADJ3 life-lost) OR (year* ADJ3 (living OR lived) ADJ3 disabilit*) OR mortalit* OR morbidit* OR sequelae* OR (cogniti* ADJ3 (defect* OR impair*)) OR (burden* ADJ3 disease*) OR dement*).ti.) NOT (exp animals/ NOT humans/) NOT (conference abstract)

Web of Science Core Collection* (1) 48

TS=(((environment* OR pollut* OR traffic* OR aircraft* OR airplane* OR airport* OR aviation* OR industr* OR annoy* OR exposure* OR occupation* OR work* OR railway* OR railroad* OR road OR transport*) NEAR/2 noise*) AND ((disease* OR develop*) NEAR/2 model*) AND (neoplas* OR cancer* OR diabet* OR coronar* OR heart OR cardiovascul* OR cerebrovascul* OR atri*-fibril* OR (disease* NEAR/2 model*) OR ((health OR mild OR moderate* OR sever* OR asymptom*) NEAR/2 (state* OR status OR outcome* OR impair*)) OR stroke* OR daly OR yll OR yld OR (disability*-adjust* NEAR/2 (life-year* OR life-year)) OR (year* NEAR/2 life-lost) OR (year* NEAR/2 (living OR lived) NEAR/2 disabilit*) OR mortalit* OR morbidit* OR sequelae* OR (cogniti* NEAR/2 (defect* OR impair*)) OR (burden* NEAR/2 disease*) OR dement*) NOT ((animals) NOT (humans))) AND DT=(Article OR Review OR Letter OR Early Access)

Web of Science Core Collection* (2) 262

TI=(((environment* OR pollut* OR traffic* OR aircraft* OR airplane* OR airport* OR aviation* OR industr* OR annoy* OR exposure* OR occupation* OR work* OR railway* OR railroad* OR road OR transport*) NEAR/2 noise*) AND (neoplas* OR cancer* OR diabet* OR coronar* OR heart OR cardiovascul* OR cerebrovascul* OR atri*-fibril* OR (disease* NEAR/2 model*) OR ((health OR mild OR moderate* OR sever* OR asymptom*) NEAR/2 (state* OR status OR outcome* OR impair*)) OR stroke* OR daly OR yll OR yld OR (disability*-adjust* NEAR/2 (life-year* OR life-year)) OR (year* NEAR/2 life-lost) OR (year* NEAR/2 (living OR lived) NEAR/2 disabilit*) OR mortalit* OR morbidit* OR sequelae* OR (cogniti* NEAR/2 (defect* OR impair*)) OR (burden* NEAR/2 disease*) OR dement*) NOT (animals NOT humans)) AND DT=(Article OR Review OR Letter OR Early Access)

Google.com

"environmental|pollution|traffic|aircraft|airplane|industrial|annoyance|exposure|occupational|railway AROUND(3) noise" disease|daly|yld file-type:pdf -pmid -doi

2. Supplementary tables and figures

Table 1: Brief lay descriptions for 82 environmental and non-environmental noise-related health states

	**Health state description**
**Cancer**	
Cancer, diagnosis and primary therapy	has pain, nausea, fatigue, weight loss and high anxiety.
Cancer, metastatic	has severe pain, extreme fatigue, weight loss and high anxiety.
Mastectomy	had one of her breasts removed and sometimes has pain or swelling in the arms.
Stoma	has a pouch attached to an opening in the belly to collect and empty stools.
Terminal phase, with medication (for cancers, end-stage kidney/liver disease)	has lost a lot of weight and regularly uses strong medication to avoid constant pain. The person has no appetite, feels nauseous, and needs to spend most of the day in bed.
Terminal phase, without medication (for cancers, end-stage kidney/liver disease)	has lost a lot of weight and has constant pain. The person has no appetite, feels nauseous, and needs to spend most of the day in bed.
**Cardiovascular and circulatory disease**	
Acute myocardial infarction: days 1-2	has severe chest pain that becomes worse with any physical activity. The person feels nauseous, short of breath, and very anxious.
Acute myocardial infarction: days 3-28	gets short of breath after heavy physical activity, and tires easily, but has no problems when at rest. The person has to take medication every day and has some anxiety.
Angina pectoris: mild	has chest pain that occurs with strenuous physical activity, such as running or lifting heavy objects. After a brief rest, the pain goes away.
Angina pectoris: moderate	has chest pain that occurs with moderate physical activity, such as walking uphill or more than half a kilometer (around a quarter-mile) on level ground. After a brief rest, the pain goes away.
Angina pectoris: severe	has chest pain that occurs with minimal physical activity, such as walking only a short distance. After a brief rest, the pain goes away. The person avoids most physical activities because of the pain.
Cardiac conduction disorders and cardiac dysrhythmias	has periods of rapid and irregular heartbeats and occasional fainting.
Claudication	has cramping pains in the legs after walking a medium distance. The pain goes away after a short rest.
Heart failure: mild	is short of breath and easily tires with moderate physical activity, such as walking uphill or more than a quarter-mile on level ground. The person feels comfortable at rest or during activities requiring less effort.

Heart failure: moderate	is short of breath and easily tires with minimal physical activity, such as walking only a short distance. The person feels comfortable at rest but avoids moderate activity.
Heart failure: severe	is short of breath and feels tired when at rest. The person avoids any physical activity, for fear of worsening the breathing problems.
Stroke: long-term consequences, mild	has some difficulty in moving around and some weakness in one hand, but is able to walk without help.
Stroke: long-term consequences, moderate	has some difficulty in moving around, and in using the hands for lifting and holding things, dressing and grooming.
Stroke: long-term consequences, moderate plus cognitive problems	has some difficulty in moving around, in using the hands for lifting and holding things, dressing and grooming, and in speaking. The person is often forgetful and confused.
Stroke: long-term consequences, severe	is confined to bed or a wheelchair, has difficulty speaking and depends on others for feeding, toileting and dressing.
Stroke: long-term consequences, severe plus cognitive problems	is confined to bed or a wheelchair, depends on others for feeding, toileting and dressing, and has difficulty speaking, thinking clearly and remembering things.
**Diabetes, digestive, and genitourinary disease**	
Diabetic foot	has a sore on the foot that is swollen and causes some difficulty in walking.
Diabetic neuropathy	has pain, tingling and numbness in the arms, legs, hands and feet. The person sometimes gets cramps and muscle weakness.
Chronic kidney disease (stage IV)	tires easily, has nausea, reduced appetite and difficulty sleeping.
End-stage renal disease: with kidney transplant	sometimes feels tired and down, and has some difficulty with daily activities.
End-stage renal disease: on dialysis	is tired and has itching, cramps, headache, joint pains and shortness of breath. The person needs intensive medical care every other day lasting about half a day.
Infertility: primary	wants to have a child and has a fertile partner, but the couple cannot conceive.
Infertility: secondary	has at least one child, and wants to have more children. The person has a fertile partner, but the couple cannot conceive.
**Chronic respiratory diseases**	
Asthma, controlled	has wheezing and cough once a month, which does not cause difficulty with daily activities.
Asthma, partially controlled	has wheezing and cough once a week, which causes some difficulty with daily activities.

COPD and other chronic respiratory disease, mild	has cough and shortness of breath after heavy physical activity, but is able to walk long distances and climb stairs.
COPD and other chronic respiratory disease, severe	has cough, wheezing and shortness of breath all the time. The person has great difficulty walking even short distances or climbing any stairs, feels tired when at rest, and is anxious.
**Neurological disorders**	
Dementia: mild	has some trouble remembering recent events, and finds it hard to concentrate and make decisions and plans.
Dementia: moderate	has memory problems and confusion, feels disoriented, at times hears voices that are not real, and needs help with some daily activities.
Dementia: severe	has complete memory loss; no longer recognizes close family members; and requires help with all daily activities.
Multiple sclerosis, severe	has slurred speech and difficulty swallowing. The person has weak arms and hands, very limited and stiff leg movement, has loss of vision in both eyes and cannot control urinating.
Epilepsy: severe (seizures ≥ once per month)	has sudden seizures one or more times each month, with violent muscle contractions and stiffness, loss of consciousness, and loss of urine or bowel control. Between seizures the person has memory loss and difficulty concentrating.
Epilepsy: less severe (seizures < once per month)	has sudden seizures two to five times a year, with violent muscle contractions and stiffness, loss of consciousness, and loss of urine or bowel control.
Parkinson's disease: mild	has mild tremors and moves a little slowly, but is able to walk and do daily activities without assistance.
Parkinson's disease: moderate	has moderate tremors and moves slowly, which causes some difficulty in walking and daily activities. The person has some trouble swallowing, talking, sleeping, and remembering things.
Parkinson's disease: severe	has severe tremors and moves very slowly, which causes great difficulty in walking and daily activities. The person falls easily and has a lot of difficulty talking, swallowing, sleeping, and remembering things.
**Mental, behavioural, and substance use disorders**	
Anxiety disorders: mild	feels mildly anxious and worried, which makes it slightly difficult to concentrate, remember things, and sleep. The person tires easily but is able to perform daily activities.
Anxiety disorders: moderate	feels anxious and worried, which makes it difficult to concentrate, remember things, and sleep. The person tires easily and finds it difficult to perform daily activities.
Anxiety disorders: severe	constantly feels very anxious and worried, which makes it difficult to concentrate, remember things and sleep. The person has lost pleasure in life and thinks about suicide.

Major depressive disorder: mild episode	feels persistent sadness and has lost interest in usual activities. The person sometimes sleeps badly, feels tired, or has trouble concentrating but still manages to function in daily life with extra effort.
Major depressive disorder: moderate episode	has constant sadness and has lost interest in usual activities. The person has some difficulty in daily life, sleeps badly, has trouble concentrating, and sometimes thinks about harming himself (or herself).
Major depressive disorder: severe episode	has overwhelming, constant sadness and cannot function in daily life. The person sometimes loses touch with reality and wants to harm or kill himself (or herself).
Attention deficit hyperactivity disorder	is hyperactive and has difficulty concentrating, remembering things, and completing tasks.
Conduct disorder	has frequent behavior problems, which are sometimes violent. The person often has difficulty interacting with other people and feels irritable.
**Hearing and vision loss**	
Hearing loss: mild	has great difficulty hearing and understanding another person talking in a noisy place (for example, on an urban street).
Hearing loss: moderate	is unable to hear and understand another person talking in a noisy place (for example, on an urban street), and has difficulty hearing another person talking even in a quiet place or on the telephone.
Hearing loss: severe	is unable to hear and understand another person talking, even in a quiet place, and unable to take part in a phone conversation. Difficulties with communicating and relating to others cause emotional impact at times (for example worry or depression).
Hearing loss: profound	is unable to hear and understand another person talking, even in a quiet place, is unable to take part in a phone conversation, and has great difficulty hearing anything in any other situation. Difficulties with communicating and relating to others often cause worry, depression or loneliness.
Hearing loss: complete	cannot hear at all in any situation, including even the loudest sounds, and cannot communicate verbally or use a phone. Difficulties with communicating and relating to others often cause worry, depression or loneliness.
Hearing loss: mild with ringing	has great difficulty hearing and understanding another person talking in a noisy place (for example, on an urban street), and sometimes has annoying ringing in the ears.
Hearing loss: moderate with ringing	is unable to hear and understand another person talking in a noisy place (for example, on an urban street), has difficulty hearing another person talking even in a quiet place or on the phone, and has annoying ringing in the ears for 5 minutes at a time, almost every day.

Hearing loss: severe with ringing	is unable to hear and understand another person talking, even in a quiet place, is unable to take part in a phone conversation, and has annoying ringing in the ears for more than 5 minutes at a time, almost every day. Difficulties with communicating and relating to others cause emotional impact at times (for example worry or depression).
Hearing loss: profound with ringing	is unable to hear and understand another person talking, even in a quiet place, is unable to take part in a phone conversation, has great difficulty hearing anything in any other situation, and has annoying ringing in the ears for more than 5 minutes at a time, several times a day. Difficulties with communicating and relating to others often cause worry, depression, or loneliness.
Hearing loss: complete with ringing	cannot hear at all in any situation, including even the loudest sounds, and cannot communicate verbally or use a phone, and has very annoying ringing in the ears for more than half of the day. Difficulties with communicating and relating to others often cause worry, depression or loneliness.
Distance vision: mild impairment	has some difficulty with distance vision, for example reading signs, but no other problems with eyesight.
Distance vision: moderate impairment	has vision problems that make it difficult to recognize faces or objects across a room.
Distance vision: severe impairment	has severe vision loss, which causes difficulty in daily activities, some emotional impact (for example worry), and some difficulty going outside the home without assistance.
Distance vision blindness	is completely blind, which causes great difficulty in some daily activities, worry and anxiety, and great difficulty going outside the home without assistance.
**Other**	
Anemia: mild	feels slightly tired and weak at times, but this does not interfere with normal daily activities.
Anemia: moderate	feels moderate fatigue, weakness, and shortness of breath after exercise, making daily activities more difficult.
Anemia: severe	feels very weak, tired and short of breath, and has problems with activities that require physical effort or deep concentration.
Annoyance: moderate	is moderately annoyed because of loud noises in the surroundings.
Annoyance: severe	is highly annoyed because of loud noises in the surroundings.
Cognitive impairments: mild	is slow in learning at school. As an adult, the person has some difficulty doing complex or unfamiliar tasks but otherwise functions independently.

Cognitive impairments: moderate	has low intelligence and is slow in learning to speak and to do simple tasks. As an adult, the person requires support to live independently and raise children and can only work at simple supervised jobs.
Cognitive impairments: severe	has very low intelligence, speaks few words, and needs constant supervision and help with all daily activities.
Generic uncomplicated disease: anxiety about diagnosis	has a disease diagnosis that causes some worry about the future. As an adult, the person has minor difficulties in daily activities.
Generic uncomplicated disease: worry and daily medication	has a chronic disease that requires medication every day and causes some worry but minimal interference with daily activities.
Intensive care unit admission	is very ill and often asleep or unconscious; when awake cannot move in bed, cannot speak, is completely dependent on others and is anxious.
Loss of smell/taste	is unable to smell or taste anything.
Motor impairment: mild	has some difficulty in moving around but is able to walk without help.
Motor impairment: moderate	has some difficulty in moving around, and difficulty in lifting and holding objects, dressing and sitting upright, but is able to walk without help.
Motor impairment: severe	is unable to move around without help, and is not able to lift or hold objects, get dressed or sit upright.
Sleep disturbance without environmental noise as the source	has difficulty falling or staying asleep
Sleep disturbance with environmental noise as the source	has difficulty falling or staying asleep because of loud noises in the surroundings
Spinal cord lesion at neck level: treated	is paralyzed from the neck down and cannot feel or move the arms and legs.
Tinnitus	has very annoying ringing in the ears for more than half of the day.

Figure 1: Distribution of gender, age groups, and educational level of the NOISE cohort sample, per country

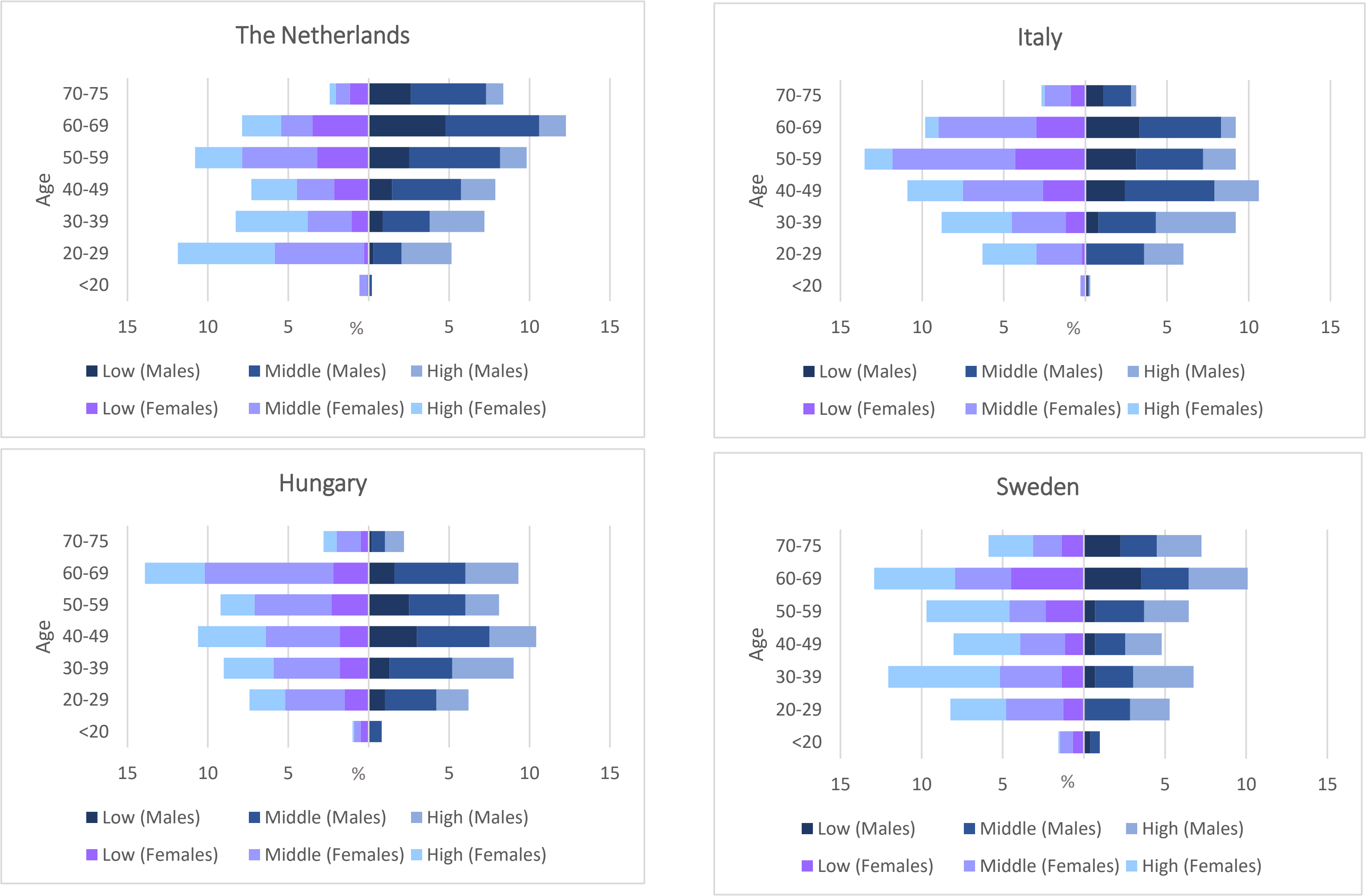

Figure 2: Distribution of gender and age groups of the NOISE cohort sample *vs*. the national population, per country

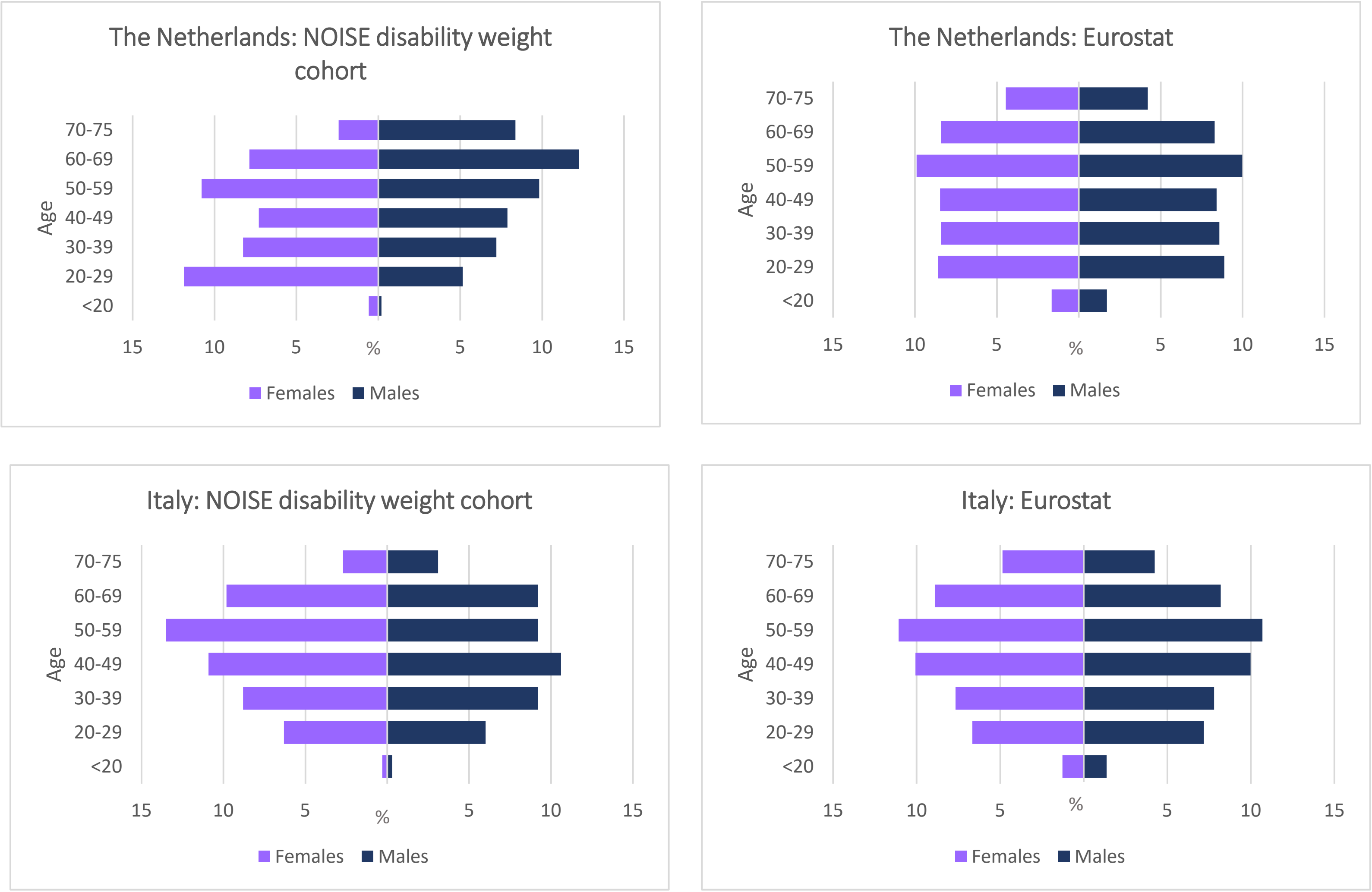

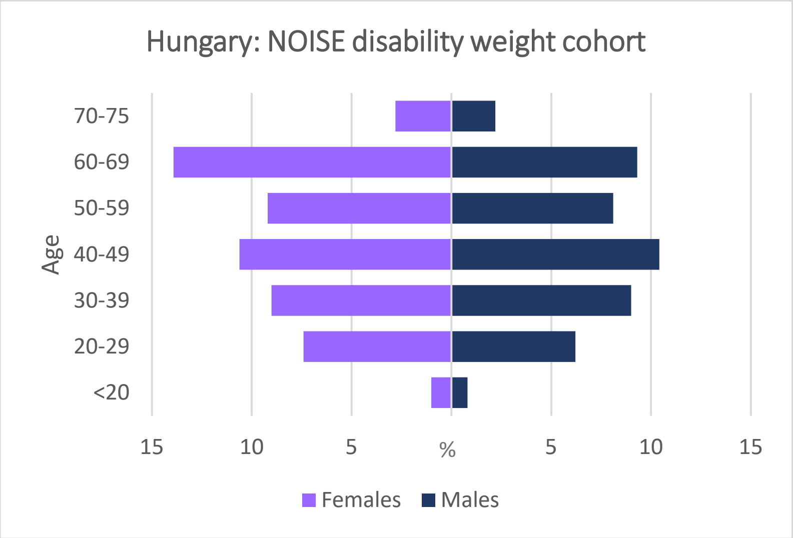
Hungary: NOISE disability weight cohort
70-75
60-69
50-59
40-49
30-39
20-29
<20
Age
15
10
5
%
5
10
15
Females
Males

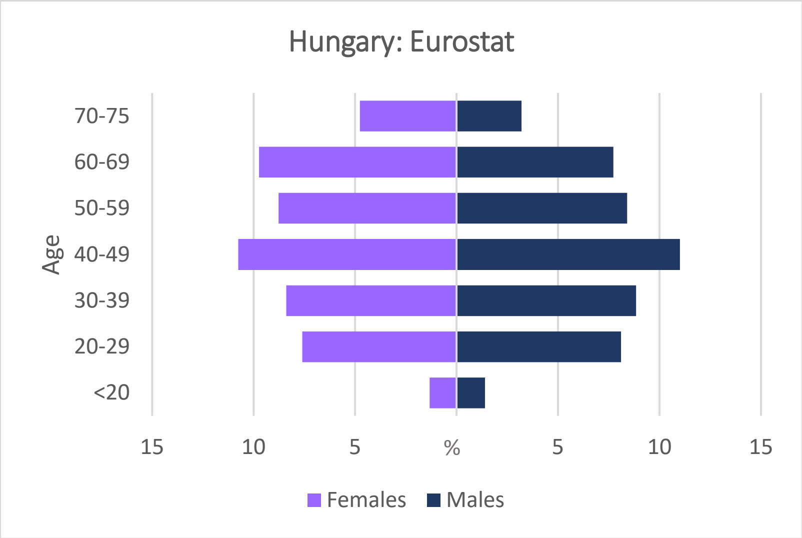
Hungary: Eurostat
70-75
60-69
50-59
40-49
30-39
20-29
<20
Age
15
10
5
%
5
10
15
Females
Males

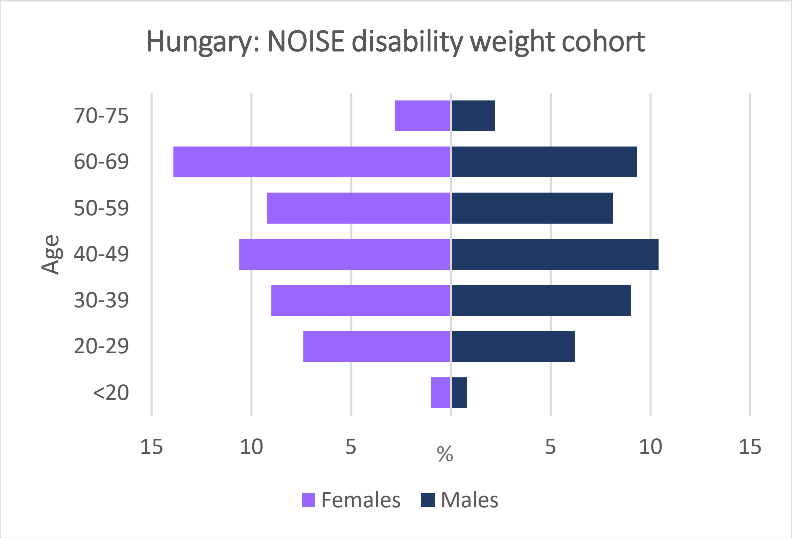
Hungary: NOISE disability weight cohort
70-75
60-69
50-59
40-49
30-39
20-29
<20
Age
15
10
5
%
5
10
15
Females
Males

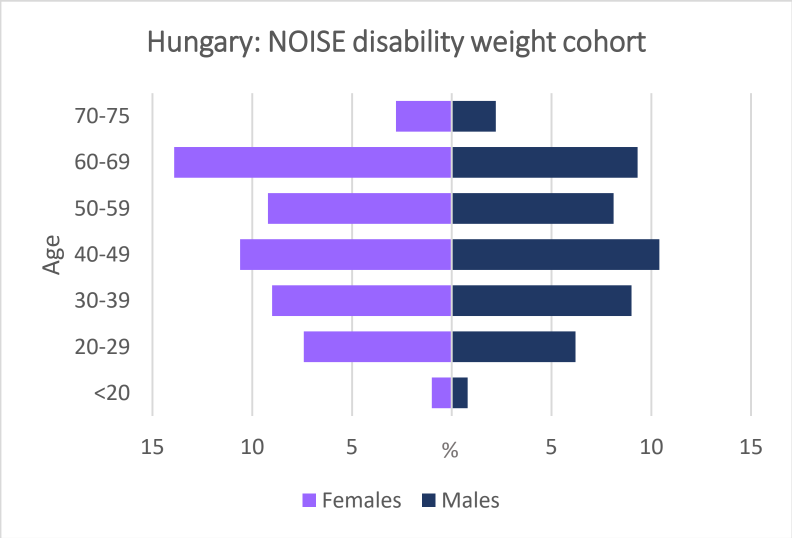
Hungary: NOISE disability weight cohort
70-75
60-69
50-59
40-49
30-39
20-29
<20
Age
15
10
5
%
5
10
15
Females
Males

Figure 3: Test re-test of the paired comparison, by country and educational level

	The Netherlands	Italy	Hungary	Sweden	Total
**Overall**	0.799	0.74	0.733	0.749	**0.756**
Same order	0.823	0.753	0.756	0.792	**0.782**
Reversed order	0.772	0.728	0.709	0.702	**0.728**
**Low educated**	0.789	0.732	0.715	0.736	0.745
Same order	0.829	0.779	0.783	0.8	0.799
Reversed order	0.754	0.681	0.66	0.676	0.695
**Middle educated**	0.798	0.742	0.728	0.74	0.752
Same order	0.824	0.741	0.751	0.767	0.771
Reversed order	0.767	0.743	0.705	0.708	0.731
**High educated**	0.806	0.745	0.752	0.763	0.767
Same order	0.817	0.752	0.753	0.809	0.787
Reversed order	0.793	0.739	0.752	0.717	0.747

Figure 4: Response probabilities for paired comparisons per country, and overall

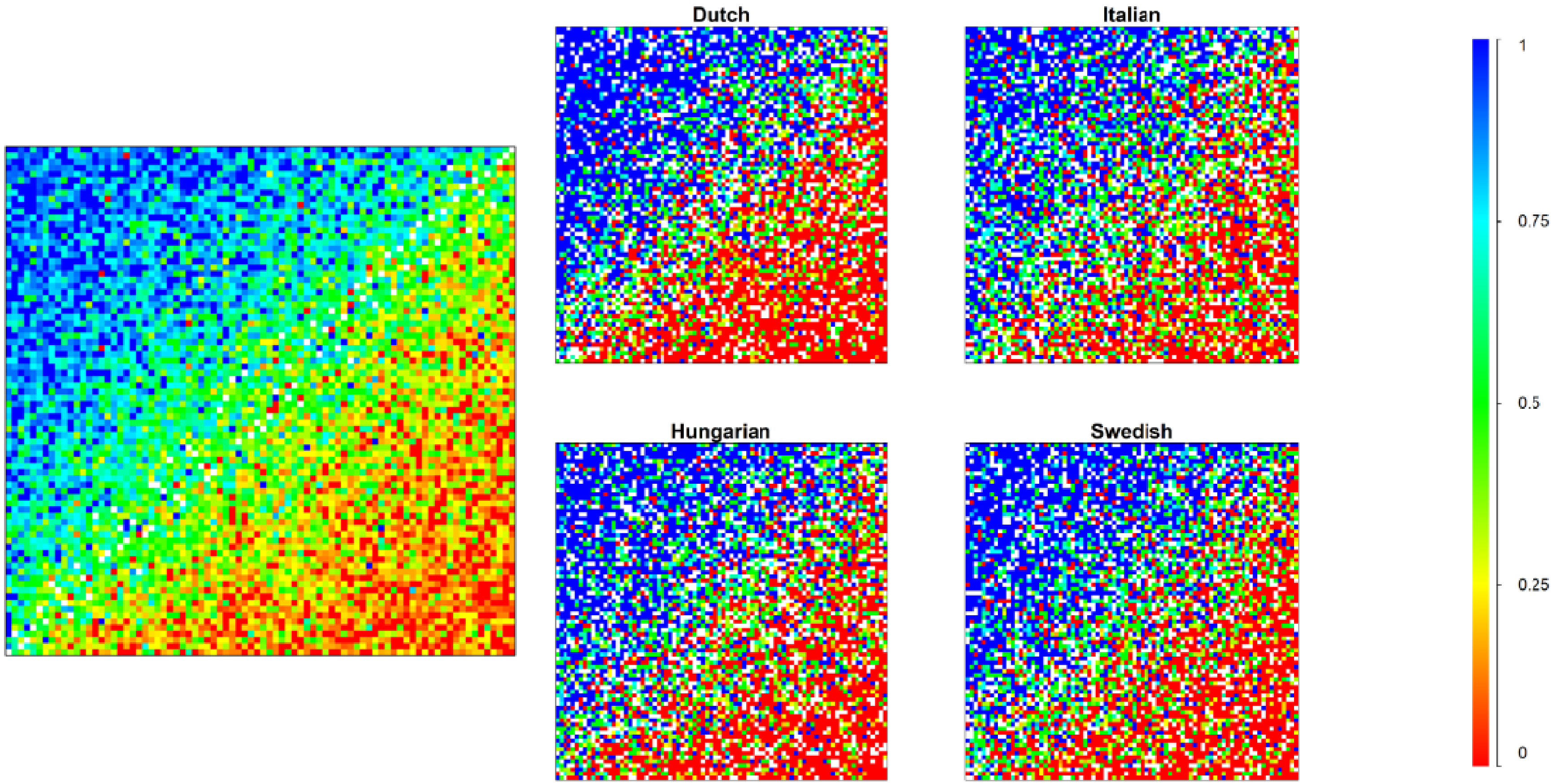

Red corresponds of less than 0.25. Orange, yellow, and green correspond to probabilities between 0.25 and 0.75. Blue corresponds to probabilities greater than 0.75. Please note that not all possible 82x82 pairs were evaluated by pairwise comparison, which is indicated by some white spaces in the figure.

Figure 5: Pearson correlation coefficients between country-specific and pooled regression analyses

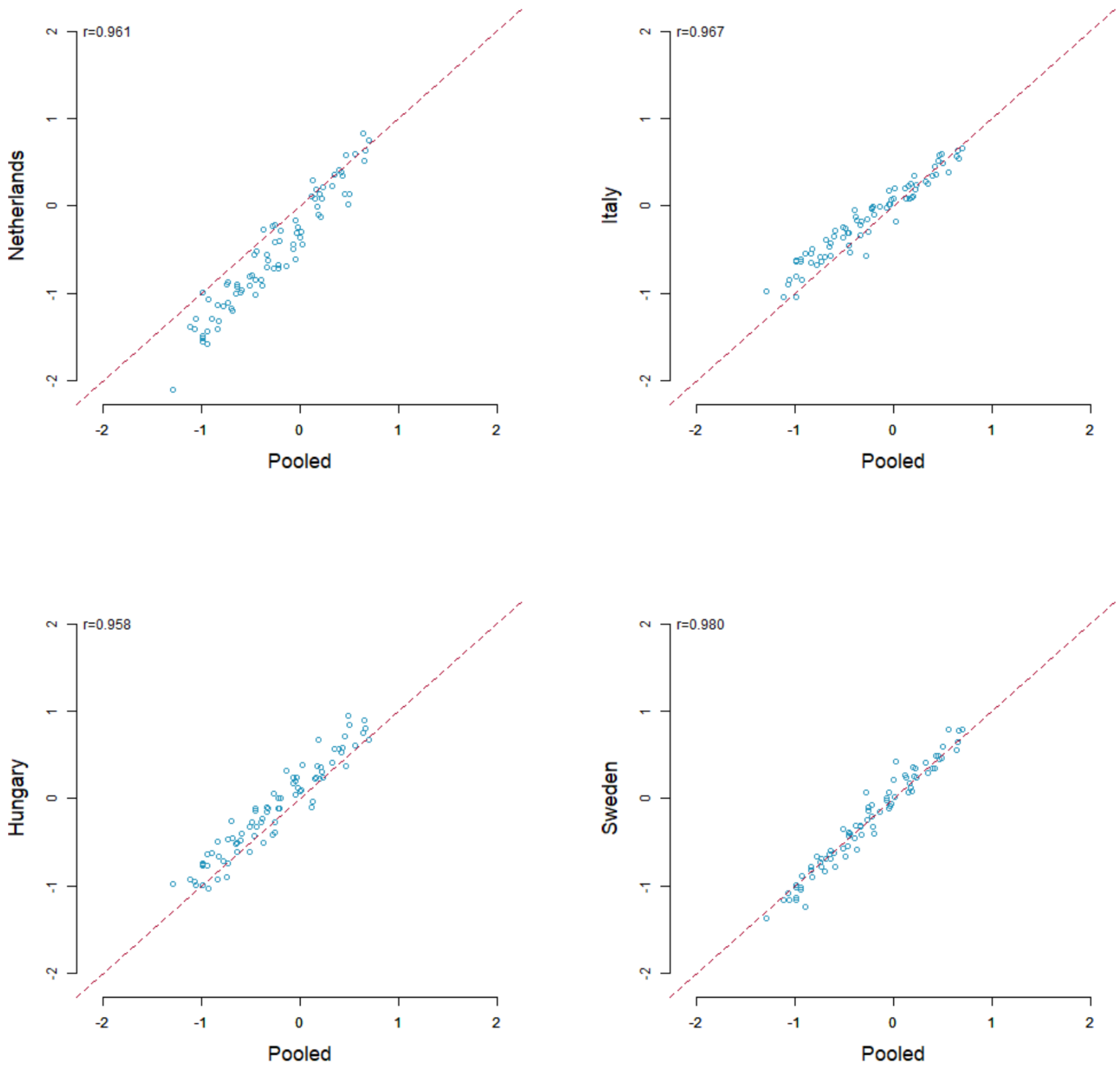

Figure 6: Pearson correlation coefficients within country-specific regression analyses

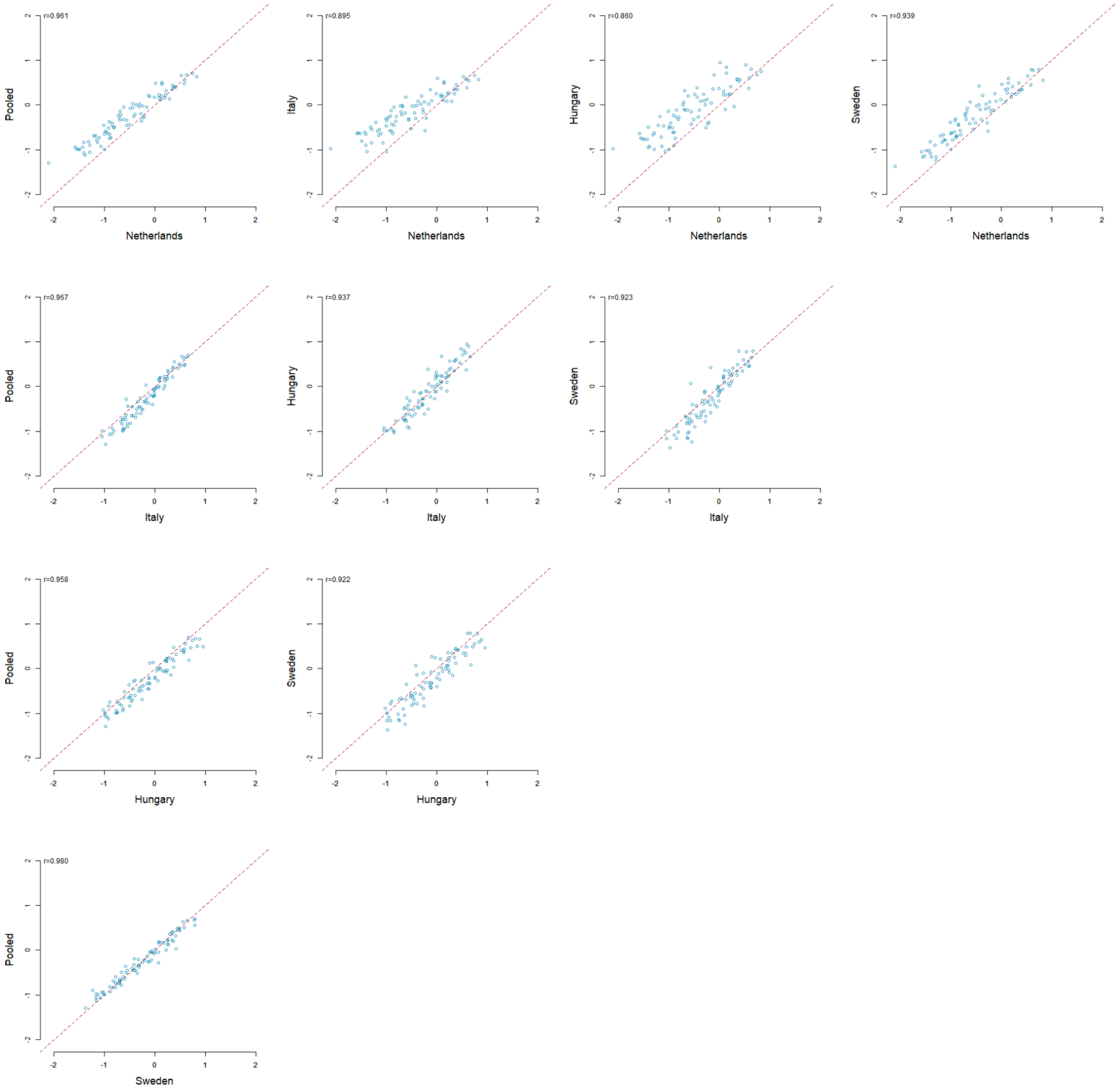

3. STROBE Statement—checklist of items that should be included in reports of observational studies

	Item No.	Recommendation	Page No.	Relevant text from manuscript
**Title and abstract**	1	(*a*) Indicate the study's design with a commonly used term in the title or the abstract	Lines 1/2, pp1	
		(*b*) Provide in the abstract an informative and balanced summary of what was done and what was found	Lines 9/31, pp 2	
**Introduction**				
Background/rationale	2	Explain the scientific background and rationale for the investigation being reported	Lines 57/93, pp 4-5	
Objectives	3	State specific objectives, including any prespecified hypotheses	Lines 95/101, pp 5	
**Methods**				
Study design	4	Present key elements of study design early in the paper	Lines 103/111, pp 6	
Setting	5	Describe the setting, locations, and relevant dates, including periods of recruitment, exposure, follow-up, and data collection	Lines 103/111, pp 6	
Participants	6	(*a*) *Cohort study*—Give the eligibility criteria, and the sources and methods of selection of participants. Describe methods of follow-up<br>*Case-control study*—Give the eligibility criteria, and the sources and methods of case ascertainment and control selection. Give the rationale for the choice of cases and controls<br>*Cross-sectional study*—Give the eligibility criteria, and the sources and methods of selection of participants	Lines 113/123, pp 6	
		(*b*) *Cohort study*—For matched studies, give matching criteria and number of exposed and unexposed<br>*Case-control study*—For matched studies, give matching criteria and the number of controls per case	N/A	
Variables	7	Clearly define all outcomes, exposures, predictors, potential confounders, and effect modifiers. Give diagnostic criteria, if applicable	N/A	
Data sources/ measurement	8*	For each variable of interest, give sources of data and details of methods of assessment (measurement). Describe comparability of assessment methods if there is more than one group	Lines 114/117, pp 6 & Line 124/145 pp 7	
Bias	9	Describe any efforts to address potential sources of bias	Lines 146/162, pp 8	
Study size	10	Explain how the study size was arrived at	Lines 119/123, pp 6	

Continued on next page

Quantitative variables	11	Explain how quantitative variables were handled in the analyses. If applicable, describe which groupings were chosen and why	Lines 163/220, pp 9/11
Statistical methods	12	(*a*) Describe all statistical methods, including those used to control for confounding	Lines 163/220, pp 9/11
		(*b*) Describe any methods used to examine subgroups and interactions	Lines 163/220, pp 9/11
		(*c*) Explain how missing data were addressed	
		(*d*) *Cohort study*—If applicable, explain how loss to follow-up was addressed<br>*Case-control study*—If applicable, explain how matching of cases and controls was addressed<br>*Cross-sectional study*—If applicable, describe analytical methods taking account of sampling strategy	Lines 163/220, pp 9/11
		(*e*) Describe any sensitivity analyses	N/A
**Results**			
Participants	13*	(a) Report numbers of individuals at each stage of study—eg numbers potentially eligible, examined for eligibility, confirmed eligible, included in the study, completing follow-up, and analysed	Lines 223/233, pp 11
		(b) Give reasons for non-participation at each stage	N/A
		(c) Consider use of a flow diagram	N/A
Descriptive data	14*	(a) Give characteristics of study participants (eg demographic, clinical, social) and information on exposures and potential confounders	Lines 223/233, pp 11
		(b) Indicate number of participants with missing data for each variable of interest	N/A
		(c) *Cohort study*—Summarise follow-up time (eg, average and total amount)	N/A
Outcome data	15*	*Cohort study*—Report numbers of outcome events or summary measures over time	N/A
		Case-control study—Report numbers in each exposure category, or summary measures of exposure	N/A
		Cross-sectional study—Report numbers of outcome events or summary measures	N/A
Main results	16	(*a*) Give unadjusted estimates and, if applicable, confounder-adjusted estimates and their precision (eg, 95% confidence interval). Make clear which confounders were adjusted for and why they were included	Lines 236/296, pp 13/17
		(*b*) Report category boundaries when continuous variables were categorized	N/A
		(*c*) If relevant, consider translating estimates of relative risk into absolute risk for a meaningful time period	N/A

Continued on next page

Other analyses	17	Report other analyses done—eg analyses of subgroups and interactions, and sensitivity analyses	Lines 236/296, pp 13/17
**Discussion**			
Key results	18	Summarise key results with reference to study objectives	Lines 299/305, pp 17
Limitations	19	Discuss limitations of the study, taking into account sources of potential bias or imprecision. Discuss both direction and magnitude of any potential bias	Lines 382/403, pp 21
Interpretation	20	Give a cautious overall interpretation of results considering objectives, limitations, multiplicity of analyses, results from similar studies, and other relevant evidence	Lines 306/380, pp 18/20
Generalisability	21	Discuss the generalisability (external validity) of the study results	Lines 329/334, pp 18/19
**Other information**			
Funding	22	Give the source of funding and the role of the funders for the present study and, if applicable, for the original study on which the present article is based	Lines 443/444, pp 23

*Give information separately for cases and controls in case-control studies and, if applicable, for exposed and unexposed groups in cohort and cross-sectional studies.

Note: An Explanation and Elaboration article discusses each checklist item and gives methodological background and published examples of transparent reporting. The STROBE checklist is best used in conjunction with this article (freely available on the Web sites of PLoS Medicine at http://www.plosmedicine.org/, Annals of Internal Medicine at http://www.annals.org/, and Epidemiology at http://www.epidem.com/). Information on the STROBE Initiative is available at www.strobe-statement.org.

4. European NOISE disability weights measurement study: questionnaire

Module 1. Demographics, Part I

Q1. **What is your age?**

Q2. **What is your gender?**

- ☐ male
- ☐ female
- ☐ other

Q3 **What is the highest level of education you completed?**

NL:

- ☐ geen of basisonderwijs
- ☐ LBO / VMBO (kader- of beroepsgericht) / MBO 1 / VBO
- ☐ MAVO / HAVO of VWO (overgegaan naar 4e klas) / VMBO (theoretisch of gemengd) / (M)ULO
- ☐ MBO 2, 3, 4 of MBO vóór 1998
- ☐ HAVO of VWO (met diploma afgerond) / HBS / MMS
- ☐ HBO propedeuse
- ☐ universitair propedeuse
- ☐ HBO bachelor (of HBO vóór 2002)
- ☐ HBO master
- ☐ universitair bachelor / kandidaats
- ☐ universitair master / doctoraal / postdoctoral

IT:

- ☐ Scuola elementare primaria
- ☐ Scuola media inferiore
- ☐ Istituto professionale
- ☐ Scuola media superiore
- ☐ Laurea breve
- ☐ Laurea Magistrale od a ciclo unico
- ☐ Dottorato di ricercar

SE:

- ☐ Grundskola, årskurs 1–6, Folkskola
- ☐ Grundskola, årskurs 7–9, Realskola, Enhetsskola, vuxenutbildning grundskolekompetens
- ☐ Gymnasieskola eller fackskola, vuxenutbildning grundskolekompetens
- ☐ Yrkesskola, flickskola
- ☐ Gymnasieskola eller fackskola, vuxenutbildning gymnasiekompetens
- ☐ Eftergymnasial utbildning utanför högskola/universitet (minst 1 år. t.ex. folkhögskola, kompletterande utbildning, kvalificerad yrkesutbildning, militära utbildningar, polisutbildning, påbyggnadsutbildning) högskola eller universitet (inklusive licenciat-/doktorsexamen på forskarutbildning)

HU:
- ☐ Általános iskola 1-3. évfolyam
- ☐ Általános iskola 4-7. évfolyam
- ☐ Általános iskola 8. évfolyam
- ☐ Szakképzettség érettségi bizonyítvány nélkül (pl. szakmunkásképző, szakiskolai bizonyítvány)
- ☐ Érettségi bizonyítvány szakképesítés nélkül (pl. gimnáziumi érettségi
- ☐ Érettségire épülő bizonyítvány szakképesítéssel (pl. érettségivel együtt szerzett szakmai végzettség)
- ☐ Érettségire épülő iskolarendszerben szerzett szakképesítés
- ☐ Felsőoktatási szakképzésben szerzett bizonyítvány
- ☐ Főiskolai vagy felsőfokú alapképzésben (BA/BSc) szerzett (vagy azzal egyenértékű) oklevél
- ☐ Egyetemi vagy felsőfokú mesterképzésben (MA/MSc) vagy osztatlan képzésben szerzett (vagy azzal egyenértékű) oklevél
- ☐ Tudományos fokozatot igazoló oklevél (PhD, DLA)

Module 2. Paired comparisons

Now we want to learn how people compare **different health problems**.

A person's health may limit how well parts of his body or his mind works. As a result, some people are not able to do all of the things in life that others may do, and some people are more severely limited than others.

Following is a series of questions about different health problems. In each question two different people will be described to you. You should imagine that these two people have the same number of years left to live, and that they will experience the health problems described for the rest of their lives. Identify which person you think **is healthier overall**, in terms of having fewer physical or mental limitations on what they can do in life.

Some of the questions may be easy to answer, while others may be harder. There are no right or wrong answers to these questions. Instead, we are interested in finding out your personal views.

Q4 **Who do you think is healthier overall, the first person or the second person?**

The first person [random 1 of 82 descriptions except the one of the second person].	o	o	The second person [random 1 of 82 descriptions except the one of the first person].

Q5 **Who do you think is healthier overall, the first person or the second person?**

The first person [random 1 of 82 descriptions except the one of the second person].	o	o	The second person [random 1 of 82 descriptions except the one of the first person].

Q6 **Who do you think is healthier overall, the first person or the second person?**

The first person [random 1 of 82 descriptions except the one of the second person].	o	o	The second person [random 1 of 82 descriptions except the one of the first person].

Q7 **Who do you think is healthier overall, the first person or the second person?**

The first person [random 1 of 82 descriptions except the one of the second person].	o	o	The second person [random 1 of 82 descriptions except the one of the first person].

Q8 **Who do you think is <u>healthier overall</u>, the first person or the second person?**

The first person [random 1 of 82 descriptions except the one of the second person].	o	o	The second person [random 1 of 82 descriptions except the one of the first person].

Q9 **Who do you think is <u>healthier overall</u>, the first person or the second person?**

The first person [random 1 of 82 descriptions except the one of the second person].	o	o	The second person [random 1 of 82 descriptions except the one of the first person].

Q10 **Who do you think is <u>healthier overall</u>, the first person or the second person?**

The first person [random 1 of 82 descriptions except the one of the second person].	o	o	The second person [random 1 of 82 descriptions except the one of the first person].

Q11 **Who do you think is <u>healthier overall</u>, the first person or the second person?**

The first person [random 1 of 82 descriptions except the one of the second person].	o	o	The second person [random 1 of 82 descriptions except the one of the first person].

Q12 **Who do you think is <u>healthier overall</u>, the first person or the second person?**

The first person [random 1 of 82 descriptions except the one of the second person].	o	o	The second person [random 1 of 82 descriptions except the one of the first person].

Q13 **Who do you think is <u>healthier overall</u>, the first person or the second person?**

The first person [random 1 of 82 descriptions except the one of the second person].	o	o	The second person [random 1 of 82 descriptions except the one of the first person].

Q14 **Who do you think is <u>healthier overall</u>, the first person or the second person?**

The first person [random 1 of 82 descriptions except the one of the second person].	o	o	The second person [random 1 of 82 descriptions except the one of the first person].

Q15 **Who do you think is <u>healthier overall</u>, the first person or the second person?**

The first person [random 1 of 82 descriptions except the one of the second person].	o	o	The second person [random 1 of 82 descriptions except the one of the first person].

//The pair shown in Q16 is the same pair as in Q5. 50% in same order and 50% in reverse order.

Q16 **Who do you think is <u>healthier overall</u>, the first person or the second person?**

The first person [same description as Q5].	o	o	The second person [same description as Q5].

Module 3. Demographics, Part II

Q17. **Thinking about the last 12 months, when you are here at home, how much does environmental noise bother, disturb, or annoy you? (e.g. noise from air traffic, road traffic, rail traffic, industrial activity)**

- ☐ not at all
- ☐ slightly
- ☐ moderately
- ☐ very
- ☐ extremely

Q18. **Which of the options below reflects your current situation best?**

- ☐ I am living alone
- ☐ I am living with a partner, without children
- ☐ I am living alone with one or more children
- ☐ I am living with a partner with one or more children
- ☐ I am living with my parents, without children
- ☐ I am living with my parents, with one or more children
- ☐ I am living with my parents and partner, with one or more children
- ☐ I am living with roommates
- ☐ Other

Q19. **Which of the options below reflects your current situation best?**

- ☐ in work: employee
- ☐ in work: self-employed
- ☐ out of work for more than 1 year
- ☐ out of work for less than 1 year
- ☐ looking after others (e.g., an ill person, a child or a parent)
- ☐ a student
- ☐ retired
- ☐ unable to work

Q20. **What is your <u>annual household</u> income from all sources?** This is a sum of all incomes, benefits, pensions and profit distributions of the members of the household. Also including income from property, alimony, child benefit, housing benefit, etc.

// Netherlands + Italy

- ☐ *Less than €14.100*
- ☐ *€14.100 to less than €36.500*
- ☐ *€36.500 to less than €43.500*
- ☐ *€43.500 to less than €73.000*
- ☐ *€73.000 or more*
- ☐ don't know / don't want to say

// Hungary

- ☐ *Less than Ft 2.100.000*
- ☐ *Ft 2.100.000 to less than Ft 4.200.000*
- ☐ *Ft 4.200.000 to less than Ft 11.000.000*
- ☐ *Ft 11.000.000 to less than Ft 13.000.000*
- ☐ *Ft 13.000.000 to less than 22.000.000*
- ☐ don't know / don't want to say

// Sweden

- ☐ *Less than Kr 25.000*
- ☐ *Kr 25.000 to less than Kr 325.000*
- ☐ *Kr 325.000 to less than Kr 387.000*
- ☐ *Kr 387.000 to less than Kr 650.000*
- ☐ *Kr 650.000 or more*
- ☐ don't know / don't want to say

Q21.

	yes	no
Have you ever experienced serious illness?	o	o
Have your immediate family ever experienced serious illness?	o	o
Have people that you cared for have ever experienced serious illness?	o	o

Q22. **Do you have any of the following chronic health complaints? Select all that apply.**

☐ asthma or chronic bronchitis
☐ heart disease
☐ consequences of a stroke
☐ diabetes
☐ chronic rheumatoid arthritis
☐ severe back complaints / arthrosis of the back
☐ painful/swollen joints of knee or hip due to arthrosis
☐ cancer
☐ memory problems due to a neurological disease / dementia
☐ memory problems due to ageing
☐ depression or anxiety disorder
☐ other chronic health complaints, namely: ....
☐ no chronic health complaints
